# KSHV and the Role of Notch Receptor Dysregulation in Disease Progression

**DOI:** 10.3390/pathogens6030034

**Published:** 2017-08-04

**Authors:** Jennifer L. DeCotiis, David M. Lukac

**Affiliations:** Department of Microbiology, Biochemistry, and Molecular Genetics, and Rutgers School of Graduate Studies, Rutgers Biomedical and Health Sciences, Rutgers University, New Jersey Medical School, 225 Warren St., ICPH E 350 C, Newark, NJ 07103, USA; Decotijl@njms.rutgers.edu

**Keywords:** KSHV, HHV8, Rta, multicentric castleman’s disease, primary effusion lymphoma, kaposi’s sarcoma, notch, RBP-Jk

## Abstract

Kaposi’s sarcoma-associated herpesvirus (KSHV) is the causative agent of two human cancers, Kaposi’s Sarcoma (KS) and primary effusion lymphoma (PEL), and a lymphoproliferation, Multicentric Castleman’s Disease (MCD). Progression to tumor development in KS is dependent upon the reactivation of the virus from its latent state. We, and others, have shown that the Replication and transcriptional activator (Rta) protein is the only viral gene product that is necessary and sufficient for viral reactivation. To induce the reactivation and transcription of viral genes, Rta forms a complex with the cellular DNA binding component of the canonical Notch signaling pathway, recombination signal binding protein for Jk (RBP-Jk). Formation of this Rta:RBP-Jk complex is necessary for viral reactivation to occur. Expression of activated Notch has been shown to be dysregulated in KSHV infected cells and to be necessary for cell growth and disease progression. Studies into the involvement of activated Notch in viral reactivation have yielded varied results. In this paper, we review the current literature regarding Notch dysregulation by KSHV and its role in viral infection and cellular pathogenesis.

## 1. Kaposi’s Sarcoma-Associated Herpesvirus

Kaposi’s sarcoma-associated herpesvirus (KSHV) or human herpesvirus 8 is a γ-herpesvirus discovered in 1994 from Kaposi’s sarcoma tissue by the identification of unique viral sequences [1]. The virus was later isolated from body cavity lymphoma tissue from patients with acquired immune deficiency syndrome [2] and from Multicentric Castleman’s disease [3]. Current treatment options for the KSHV-associated diseases include anti-retroviral therapy, radiation, and in the case of Kaposi’s Sarcoma (KS) when one or a few lesions are present, surgery [4,5]. However, none of these treatments specifically target KSHV.

Recent studies have implicated the manipulation of the Notch signaling pathway in KSHV and primary effusion lymphoma (PEL) pathogenesis [6,7], adding these viral cancers to a long list of non-viral human malignancies whose etiologies are associated with dysregulated Notch (reviewed in [8,9]). Specifically, the dysregulation of recombination signal binding protein for Jk (RBP-Jk), a Notch pathway component and transcriptional regulator, has proven to be essential in the switch from latency to lytic reactivation. Other components of the Notch signaling pathway, such as the Notch receptor itself, have been shown to be dysregulated in KSHV-infected cells as well [6,10]. In fact, the activated form of the Notch receptor has a major role in KSHV-related oncogenesis [6,11,12]. However, it is not as clear what role activated Notch plays in viral reactivation, as various reports have suggested contradictory effects of activated Notch [13,14,15,16].

### 1.1. Virion Structure

**Figure 1:**
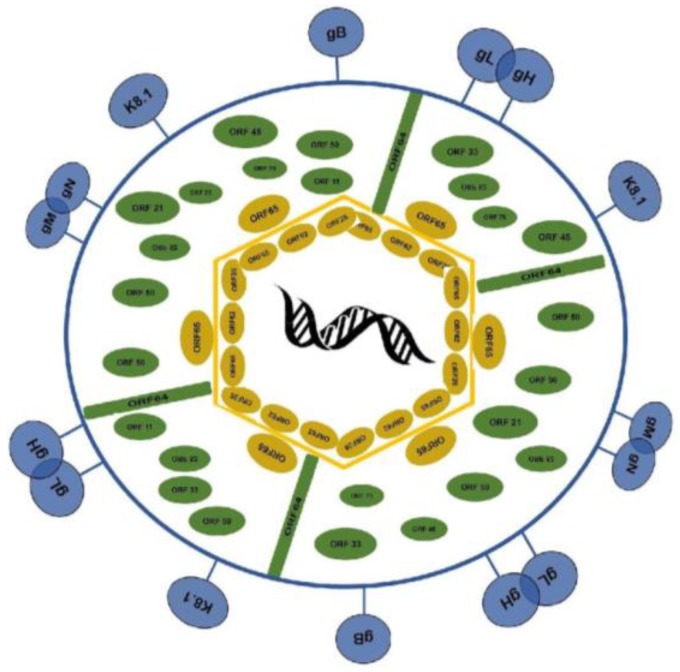
Mature Kaposi’s sarcoma-associated herpesvirus (KSHV) virion structure. The 160–170 kDa linear double stranded DNA genome is surrounded by an icosahedral capsid. This capsid is composed of viral proteins ORF62, 26, 65, and decorated with ORF25 proteins (gold ovals). The capsid is connected to the envelope (blue) via the tegument layer, which contains the viral proteins ORF 11, 21, 33, 45, 50, 52, 63, 75, (green ovals) and 64 (green rectangles). The envelope consists of a lipid bilayer decorated with viral glycoproteins (gM, gN, gL, gH, gB, K8.1 (blue lollipops)) used for the binding and entry of target cells.

KSHV belongs to the rhadinovirus genus of the herpesviridae family of viruses. Similarly to all other members of this genus, the KSHV virion is enveloped with an icosahedral capsid protecting the genome (Figure 1). The genome encodes at least 81 open reading frames (ORFs), from which all viral proteins, including those composing the mature virion, and 12 pre-microRNAs are expressed [17,18].

The KSHV envelope consists of a lipid bilayer studded with viral glycoproteins. These viral glycoproteins play a role in viral recognition of host cells and entry. The glycoprotein gB interacts with the cellular receptor integrin α3β1 to mediate viral entry [19], while K8.1, a protein commonly used as a marker of viral reactivation, interacts with host cells by binding heparan sulfate-like moieties [20].

The envelope is connected to the capsid via cellular and viral proteins organized in an intricate network of protein–protein interactions [21,22]. This network is known as the tegument layer, and is acquired by the viral capsid in the host cell cytoplasm during virion assembly and release. Tegument proteins predominantly play roles in viral release, but are also involved in immune evasion and other regulatory functions [23]. Recent studies suggest that the KSHV miRNAs are also contained within the tegument layer [24].

Tegument proteins regulate viral immune evasion by inhibiting: type I interferon (IFN)-mediated immune responses [25,26], activated caspase 1, and interleukin induction [27]. Furthermore, tegument proteins inhibit NF-κB activation [28] and purine biosynthesis [29].

As previously mentioned, this tegument layer is connected to the viral capsid. Similarly to α and β herpesviruses, KSHV’s capsid is arranged in a T = 16 icosahedral lattice with an identical inner radius to that of HSV-1 [30]. Within this capsid is a 160–170 kb linear double stranded DNA genome [31]. Approximately 140.5 kb of the genome consists mainly of the coding region unique to KSHV, and is flanked by about 35 kb of terminal repeats. Interestingly, 81 consensus and additional non-consensus RBP-Jk DNA binding sites have been identified within the KSHV genome [32] (Lukac Lab, Unpublished observations), suggesting that KSHV might manipulate the host Notch transcriptional pathway to regulate the expression of its own genes.

### 1.2. KSHV Infection of Target Cells and Egress

**Figure 2:**
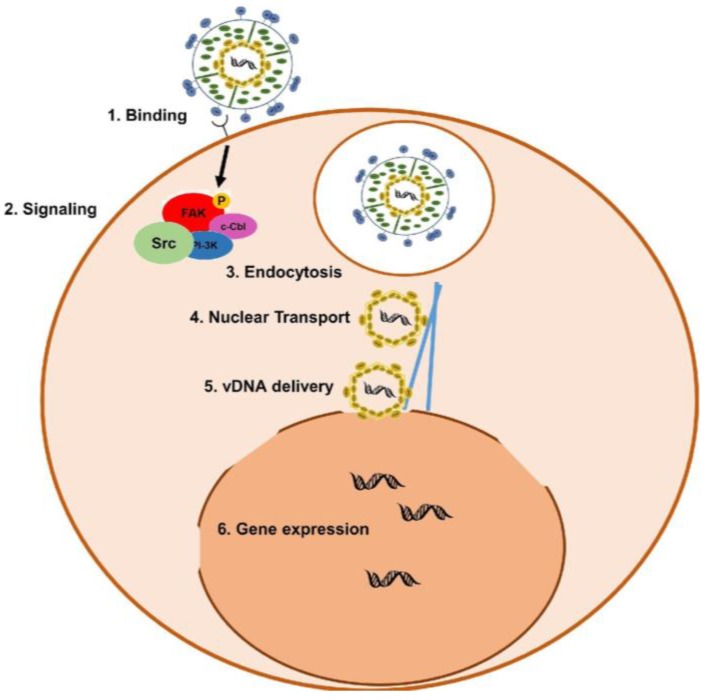
KSHV infection of target cells. Glycoproteins on the surface of the virion bind to heparan sulfate, dendritic cell specific intercellular adhesion molecule 3-grabbing nonintegrin (DC-SIGN), and integrin receptors on the surface of host cells. This interaction between the virus and the host induces a signaling cascade that recruits proteins, such as myosin and dynein (blue lines), that are required for endocytosis and the transport of viral DNA to the nucleus. Once delivered to the nucleus, viral and host cell genes are expressed to help subvert host immunity and establish latent infection.

KSHV identifies and infects cells through the use of viral glycoproteins that are found on the envelope surface (Figure 2). The binding of KSHV glycoproteins to heparan sulfates [19,33,34], integrins [35], the ephrin receptor tyrosine kinase, EphA2 [36], and dendritic cell specific intercellular adhesion molecule 3-grabbing nonintegrin (DC-SIGN) [37] not only provides the virus with an initial point of cellular contact, but also induces signal cascades involved in viral endocytosis, movement through the cytoplasm, and delivery to the nucleus [38,39].

Viral entry is facilitated by the cellular E3 ligase, c-Cbl (Casitas B-lineage Lymphoma), which translocates extracellular KSHV along with the host’s cell receptors to lipid rafts to promote the association of integrins with ephrin receptors. This results in the formation of a complex between integrins, c-Cbl, and myosin, which induces the creation of endocytic blebs [40], allowing the virus to enter the cell. KSHV binding to integrins also induces the autophosphorylation of focal adhesion kinase (FAK) which is capable of interacting with downstream effectors Src, PI3-K, and c-Cbl [41,42]. Phosphorylated FAK is also involved in endocytosis [43], to help the virus enter the cell, and associates with RhoA and cytoskeletal proteins to facilitate the transportation of the virus to the nucleus [41]. RhoA mediates the acetylation and aggregation of microtubules [44]. The microtubules and dynein motor proteins transport the virus through the cytoplasm, and subsequently deliver the virus to the nucleus [45].

Once viral DNA has entered the host cell nucleus via the nuclear pore, the genome expresses viral lytic genes involved in host cell immune evasion [46]. For instance, K5 is expressed, which downregulates the expression of cell surface molecules, helping the infected cell evade the immune system, while the expression of K2, K4, K6, and K7 has anti-apoptotic properties and protects the virus from interferons and natural killer cells [38]. At the same time, KSHV also reprograms the host cell transcriptional machinery to regulate apoptosis, the cell cycle, angiogenesis, signaling pathways, and the response to inflammation [47]. Expression of the viral lytic transcription factor, Replication and transcriptional activator (Rta), immediately following KSHV infection is believed to be involved in host cell reprogramming and the induction of the anti-apoptotic lytic genes [46]. Furthermore, Rta transactivates the lytic latency-associated nuclear antigen (LANA) promoter, indicating that the regulator of viral reactivation is also a main contributor to the establishment of latency [48]. LANA is required for the establishment and maintenance of latency [49,50] (see latent vs. lytic section). This regulation of LANA by Rta requires RBP-Jk, the DNA binding component of the Notch signaling pathway [51]. The removal of the RBP-Jk site located within the LANA promoter delays the establishment of latency post-infection, and decreases viral infectivity and genome maintenance within infected cells. It is evident that the dysregulation of the Notch signaling pathway, specifically through viral use of the RBP-Jk component, is essential for successful KSHV infection.

Upon entrance into the latent phase, the linear genome is circularized into an episome and tethered to the host cell genome [52]. The viral genome will now replicate along with the host cell genome, producing no new virus, until an environmental trigger induces the virus to switch back to its lytic state [53]. This switch is termed reactivation.

**Figure 3:**
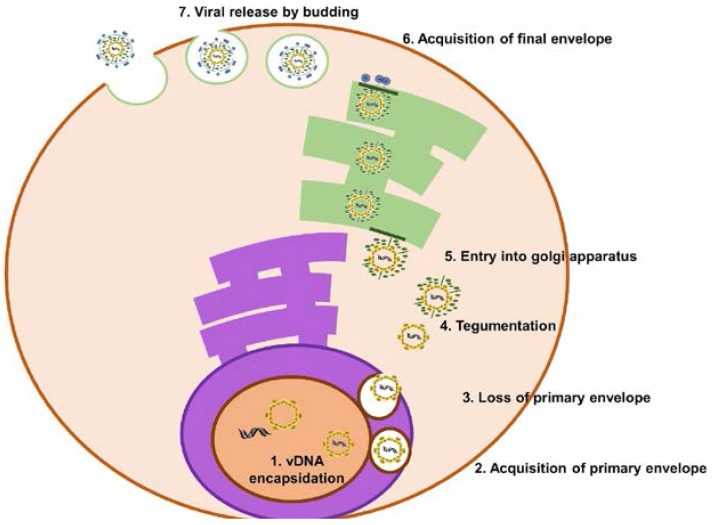
KSHV egress. Viral DNA is packaged into preformed capsid. At the inner nuclear membrane (brown), the virus acquires a primary envelope which is lost during fusion with the outer nuclear membrane (dark purple). As the encapsidated viral DNA moves through the cytoplasm it acquires tegument proteins which allow for entry into the Golgi apparatus (green). The virus acquires its final envelope by budding into Golgi-derived vesicles. These vesicles fuse with the plasma membrane, releasing the mature virion into the extracellular space.

Once the virus switches to its lytic state, and mature virions are produced, the virus leaves infected cells via exocytosis (Figure 3). During exocytosis, the virion matures by acquiring tegument proteins and the viral envelope as it passes through the nucleoplasm, cytoplasm, and Golgi apparatus [22]. Multiple models have been proposed to describe how herpesviruses mature and are released; however, evidence suggests the envelopment-deenvelopment-reenvelopment model to be the model by which maturation most likely occurs [54].

In this model, viral DNA is packaged into preformed capsids in the nucleus [22,55]. This is termed the “nucleocapsid”, which traverses the nucleoplasm via actin filaments [56], making its way to the inner nuclear membrane from which it buds, acquiring a primary envelope and tegument layer in the process [22,57,58]. This primary envelope is then lost when it fuses with the outer leaflet of the nuclear or endoplasmic membrane, thus releasing the nucleocapsids into the cytoplasm [22,55].

In the cytoplasm, the nucleocapsid acquires both its final tegument and final envelope [22,55]. This event is driven by an intricate network of protein–protein interactions [59]. Once the virus has acquired its final envelope by budding from the Golgi, it is released into the extracellular space via exocytosis. The mature virions are released when the Golgi vesicles fuse with the plasma membrane of the cell, but the proteins involved in this process have yet to be identified [22].

### 1.3. Pathogenesis

It is believed that the principal site of KSHV latency is in B lymphocytes due to the isolation of the virus from the bone marrow of infected individuals [60,61] and the detection of the virus in the CD19+ cells of KS patients [62,63]. This notion is further supported by the fact that B cells are the latency reservoirs for the closely related gammaherpesviruses Epstein–Barr virus (EBV) [64] and MHV68 [65]. The reactivation of KSHV leads to increased viral detection in the peripheral blood. It is believed that reactivation facilitates the access of KSHV to endothelial cells, where it can establish infection and initiate the development of KS. Indeed, epidemiologic data show that 55% of healthy patients with detectable KSHV in their blood progressed to KS, and 50% of human immunodeficiency virus (HIV)-infected patients progress to KS within 3.5 years of KSHV detection in their blood as compared to either healthy blood donors or HIV positive patients not infected with KSHV [66]. Furthermore, studies of antiherpesvirus drugs such as cidofovir and ganciclovir have shown a correlation between a decrease in viremia and a decrease in the detection of KSHV markers within biopsies [67,68]. Detailed pathogenic studies are hampered by the lack of a small animal model for KS that supports robust KSHV infection.

### 1.4. Transmission

There is still some debate as to how KSHV is transmitted from one individual to another. The disagreement arises in part from variations in the prevalence of KSHV and KS incidence based on age, sex, and geographical region. It is generally agreed upon that saliva [69,70] is likely the most predominant vehicle of KSHV transmission, but that there is potential for spread via blood transfusions [71], transplants [61], vertical transmission from mother to child, and sexual fluids [72]. Indeed, the oral inoculation of two marmosets led to the formation of KS-like lesions in only one of them [73].

### 1.5. Disease

As previously mentioned, KSHV is the causative agent of two human cancers, Kaposi’s sarcoma [1] and Multicentric Castleman’s disease (MCD) [3], and a lymphoproliferation, primary effusion lymphoma (PEL) [2]. 

There are an estimated 1863 to 2050 expected cases of Castleman’s disease in the U.S. per year, with 294 occurring among individuals infected with HIV [74]. Castleman’s disease patients present with non-cancerous growths in lymph tissues with approximately 70% of all cases occurring in the chest, 15% occurring in the neck, and another 15% occurring in the abdomen and pelvis [75]. Symptoms include fever, weight loss, respiratory symptoms, enlargement of both the spleen and liver, swelling of the lymph nodes, and abnormal accumulation of fluid beneath the skin [76]. At diagnosis, individuals with KSHV-associated MCD generally receive a poor prognosis [75]. Highly active antiretroviral therapy (HAART) is used in patients with acquired immunodeficiency syndrome (AIDS)-KSHV associated MCD. While effective, this therapy often diminishes the patient’s immune system and does not prevent relapse [76]. 

A condition similar to MCD, KSHV inflammatory cytokine syndrome (KICS), was recently identified. Patients with KICS exhibit similar symptoms as those with MCD, but no pathologic nodal changes are observed, nor is lymphadenopathy prominent [77]. Because a diagnosis of KICS is dependent on the exclusion of MCD diagnosis and KICS often leads to MCD development, there has been some controversy as to whether it can, in fact, be considered a distinct syndrome [78,79].

KSHV infection is not uniformly associated with MCD development, however KSHV is the causative agent of all cases of PEL. Malignant PEL cells are always infected with KSHV [80], and commonly, but not always, co-infected with EBV. This lymphoma presents as a lymphomatous growth in a liquid phase in body cavities near the heart, lungs, and the abdomen [81]. The symptoms of PEL are the result of tumor development, and patients often present with shortness of breath, enlargement of the abdomen, and an increase in abdominal pressure. Like MCD, the prognosis for those with PEL is poor, with an average survival time at diagnosis of six months even with treatment [81]. Currently only two treatment options are available for this illness because of its rarity in the general population: HAART and combination chemotherapy [81].

KSHV also causes KS, the disease in which the virus was discovered and identified as a separate entity from HIV [1]. The lesions that characterize this disease are composed of vascular spaces made up of endothelial cells that protrude into the lumen of the blood and lymphatic vessels. Since Moritz Kaposi first identified these lesions in the 19th century, four clinical forms of KS have been identified: classic KS, endemic KS, transplant-associated KS, and AIDS-associated KS [4,5].

Classic KS predominantly occurs in older, HIV-negative men of Mediterranean and Eastern European decent [82]. Because this form of KS causes little to no pain and is slow growing, aggressive therapy is not required. Surgical removal and radiotherapy are the most common treatment options for classic KS when treatment is pursued. However, because of its relatively benign nature, individuals with classic KS may choose not to undergo treatment [4,5].

Unlike classic KS, endemic or African KS is very aggressive, is regularly seen in HIV-seronegative children and young adults, and is a leading cause of death in Sub-Saharan Africa [83]. Individuals with this form of KS can develop lesions on both the skin and internal organs, the latter of which can be fatal [84]. Patients also commonly present with swollen lymph nodes throughout the body. When available, cytotoxic chemotherapy and/or radiation are used to treat this illness. In cases where KS is localized to the limbs, amputation is the preferred method of treatment [4,5].

Transplant-associated or iatrogenic KS is found in patients seropositive for KSHV taking immunosuppressant therapy. This form of KS causes a significant number of deaths in countries with high KSHV seroprevalences [85]. Individuals receiving solid organ transplants, especially liver transplants, are at highest risk for contracting this form of KS [86]. KS in these patients can be traced to the receipt of KSHV infected organs, or, most commonly, to seropositivity before transplantation and subsequent treatment with immunosuppressants. Tapering the immune-suppressive regimen is the therapy most often used to treat this form of KS [87], although the use of antiretroviral drugs has also become a treatment option [4,5].

The most aggressive and common variant of KS is AIDS-associated or epidemic KS [88]. This variant is an AIDS-defining disease and has an increased frequency in HIV positive men [89]. In more than half of those with AIDS-KS, lesions will present on internal organs causing symptoms such as diarrhea, weight loss, bleeding, cough, and even death. Treatment with HAART is the first line of defense in AIDS-KS, but is not always effective and in some cases can make KS worse [90]. If this occurs, systemic chemotherapy may be used. Although few in the general population in the U.S. are at risk for disease if exposed to KSHV, in those at risk the effects can be both painful and deadly.

### 1.6. Lytic vs. Latent Infection

KSHV contributes to KS progression using two molecular phases: latent and lytic. While the majority of infected cells within a KS tumor are latently infected [91], the expression of viral lytic genes promotes KS development (reviewed in [53]). These observations suggest that both the latent and lytic phases of KSHV are essential for the persistence of the virus and KS pathogenesis [17,53]. In transgenic mice, expression of the lytic gene vGPCR was found to be required for tumor formation and growth [92], further indicating the necessity of the lytic phase for disease progression [93]. Interestingly, the vGPCR promoter contains two RBP-Jk elements that bind to RBP-Jk and are required for Rta transactivation [94]. These observations support the direct connection between the manipulation of Notch signaling by the virus and KSHV pathogenesis.

**Figure 4:**
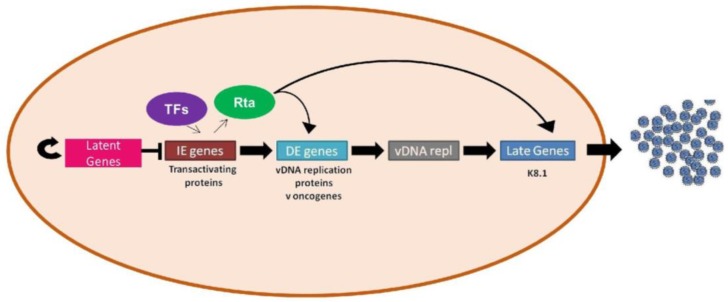
During the latent phase of the KSHV life cycle, a subset of viral genes (pink box) are expressed which maintain latency and allow the virus to subvert the host immune system. Environmental triggers induce reactivation, or the transition from the latent to lytic phase. The lytic phase is induced by cellular transcription factors (TFs, purple circle), which contribute to transcription of viral immediately early genes (maroon box), such as replication and transcriptional activator (Rta, green circle). Rta then induces the transcription of viral delayed early genes (light blue box) known to contribute to viral oncogenesis and viral DNA replication. Expression of the delayed early genes induces viral replication (grey box), which, once complete, triggers the expression of late genes (dark blue box). These late genes are involved in viral packaging. One of these late genes, K8.1, is a glycoprotein that is often used as a marker of viral reactivation. Mature virions are then released from the cell via egress as described previously (Figure 3). IE, immediate early; DE, delayed early.

During latent, or non-productive, KSHV infection, the 160–170 kb viral DNA genome [31] replicates along with the host cell genome [95], and a small subset of viral genes are expressed to maintain the viral genome and subvert the host’s immune system [96] (Figure 4). These viral genes are located in the region termed the latency locus, located between ORF69 and K14 (reviewed in [53]). Transcripts from this region include: LANA (latency-associated nuclear antigen) encoded by ORF73, v-Cyclin encoded by ORF72, v-FLIP encoded by ORF71, Kaposin encoded by K12, and 12 pre-microRNAs [53,97].

LANA, the most thoroughly investigated latency protein, is responsible for maintaining latency by inhibiting lytic gene expression [49,50] and ensuring the maintenance of the viral episome [95,98]. LANA inhibits lytic gene expression by recruiting DNA methyl transferases to lytic gene promoters, thus inhibiting the transactivation of these promoters. It is in this manner that LANA inhibits the transcription of the lytic switch protein, Rta [49]. LANA is also capable of interfering with transactivation by Rta, as LANA has been shown to interact directly with Rta [99] and associate with the cellular component of the Rta transactivation complex, RBP-Jk [100]. LANA’s interaction with RBP-Jk represses the transactivation of the Rta promoter, as mutations to RBP-Jk binding sites within the Rta promoter induce both a decrease in Rta expression and KSHV genomes [101]. Direct interaction between LANA and RBP-Jk is required for LANA to repress Rta expression and prevent uncontrolled viral reactivation [102]. As was observed when LANA expression was not properly induced in *de novo* infection, LANA mutants with defective RBP-Jk interactions fail to support episome persistence. These observations highlight the importance of Notch dysregulation by LANA in controlling latency and lytic reactivation. Another viral protein, vIRF4, also binds and sequesters RBP-Jk to hinder Notch signaling [103], with an unknown effect on viral replication. To maintain episomal KSHV, LANA tethers KSHV DNA to the host chromosome, ensuring proper segregation into daughter cells [104]. LANA is also sufficient for the induction of episomal replication, suggesting that LANA recruits cellular DNA replication machinery to the KSHV origin of replication [105]. In addition to maintaining latency, LANA also contributes to infected cell growth and survival by inhibiting the tumor suppressors p53 and pRb [106,107] and activating the oncogene, c-Myc [108].

The latent protein vCyclin is also capable of contributing to cell proliferation by constitutively activating CDK6, which phosphorylates cellular proteins such as histones and pRb [109,110]. Similarly to vCyclin, vFLIP contributes to cell survival by activating the NF-κb pathway, thus inhibiting apoptosis [111] and directly inhibiting autophagy [112,113]. The three Kaposins, A, B, and C, also affect cell signaling, potentially contributing to cell growth [114,115].

The microRNAs encoded within the intron between vFLIP and kaposin and in the coding region of ORFK12 [18] all contribute to latency by targeting either the expression of viral lytic genes or cellular proteins involved in apoptosis [116]. In addition, some microRNAs are capable of decreasing the expression of proinflammatory cytokines, thus contributing to virus escaping detection by the host’s immune system [117].

Many environmental changes can cause the virus to switch from its latent to its lytic state. These environmental changes include hypoxia, inflammation, infection by HIV, and oxidative stress [118,119,120]. In fact, hypoxia impacts KSHV reactivation in a Notch-dependent fashion, as hypoxia-inducible factor 1α (HIF-1α) coordinates with RBP-Jk to transactivate lytic genes containing HIF-1α responsive elements [121].

In addition to environmental signals, histone deacetylase inhibitors such as sodium butyrate, trichostatin A, and valproic acid reactivate KSHV from latency by inducing histone acetylation and demethylation of viral DNA [122,123,124]. Furthermore, the phorbol ester, TPA, reactivates KSHV through the mitogen-activated protein kinase (MAPK)/extracellular signal-regulated kinase (ERK) pathway [125]. Cross-linking of B cell receptors with anti-IgM antibody has also been shown to activate full lytic replication [126,127,128].

The Rta protein, encoded by ORF50, is the only viral protein that is necessary and sufficient for the induction of viral reactivation [129], thus inducing active viral replication [130]. Presumably, Rta is a key pathogenic factor, since its expression leads to the release of mature virions with the potential to infect endothelial cells and cause KS [131]. However, in order to successfully make and release mature virions, viral gene expression must occur in a time-controlled and tightly regulated manner [53] (Figure 4). Immediate early genes (IE) are the first genes expressed in lytic induction, and are involved in gene transcription and modulating cellular gene expression, preparing the host cell for viral replication. The immediate early genes include: Rta, ORF45, K8α, K8.2, K4.1, K4, ORF48, ORF29b, K3, and ORF70 [131,132].

The delayed early genes (DE) are first expressed 10–24 h post lytic induction [131,132]. These delayed early genes include: ORF57, K-bZIP, ORF59, and ORF6. ORF59 and ORF6 protein are both directly involved in viral DNA replication [133,134]. Mta, the protein encoded by ORF57, on the other hand, contributes to viral replication in a less direct manner.

Studies from our lab indicate that the presence of Mta enhances reactivation by Rta and that the expression of Mta is tightly associated with complete viral reactivation [135]. Furthermore, the knockout of Mta has been shown to disrupt the expression of lytic genes ORF59, K-bZIP, and K8.1, and decrease the amount of virus produced [136]. These results are likely due to Mta’s ability to cooperate with Rta to induce the transcription of certain viral genes in a cell line specific manner, increase RNA stability [137,138], function as a viral splicing factor [139], and promote protein translation [140,141]. Mta alone has also been shown to transactivate the viral gene product nuclear transcript-1/polyadenylated nuclear RNA (nut-1/PAN) in 293 cells without Rta, and contribute to nut-1/PAN accumulation [135]. Nut-1/PAN is a long non-coding RNA that contributes to the modulation of immune response [142].

Like Mta, K-bZIP cooperates with Rta to activate lytic promoters while also activating some promoters alone [143]. Conversely, K-bZIP has also been shown to repress Rta-mediated transactivation, suggesting that cooperation is promoter-specific [144]. K-bZIP also directly contributes to viral DNA replication by overcoming LANA repression of the OriLyt [145]. As such, K-bZIP has two roles in lytic reactivation, one as a regulator of viral gene expression, and a second as a direct facilitator of replication [97,131].

Upon the completion of viral DNA replication, late genes are expressed. These genes are expressed 48–72 h post lytic induction, and encode proteins responsible for the packaging of viral DNA [132]. These proteins make up the capsid, tegument, and glycoprotein components of the mature virion (reviewed in [97,131]).

## 2. KSHV Gene Regulation by Replication and Transcriptional Activator (Rta) Partially Mimics the Cellular Notch Protein

As mentioned, Rta is the viral gene responsible for the switch from non-productive to productive viral infection [130,146]. In performing this role, Rta initiates the transcription of lytic genes by interacting with RBP-Jk [147]. As such, Rta and activated Notch appear to be functional homologs. This 120 kDa protein is encoded by the ORF50 gene of KSHV. Its mRNA transcript is detectable within 1 h of TPA treatment, and is classified as an immediate early gene due to its insensitivity to the protein synthesis inhibitor, cycloheximide [146,148]. Rta protein is easily detectable within 8 h post induction [132].

### 2.1. Structure

Currently, no three-dimensional or high resolution structures of Rta exist. However, secondary structure predictions of Rta provide insight into its function, clearly suggesting Rta’s role as a transcriptional activator. Two nuclear localization signals are found within Rta, indicating that Rta primarily functions within the nucleus. However, only one of these nuclear localization signals has been shown to be functional [149]. This observation was confirmed via immunofluorescence of reactivation-induced infected B cells [146]. Furthermore, electrophoretic mobility shift assays using probes designed from the Mta, K-bZIP, and nut-1/PAN promoters clearly demonstrate Rta’s ability to bind DNA. Truncated Rta extending from amino acids 525–691 or amino acids 1–272 were generated and their abilities to bind to consensus responsive elements within the Mta promoter were determined by EMSA. A shift was only observed in samples incubated with the truncated Rta-containing amino acids 1–272, indicating that this N terminal region of Rta is required for DNA binding [150].

Prior to determining the Rta DNA binding domain and the cognate protein’s ability to activate the transcription of Mta and K-bZIP [150], studies showed that the C terminal transactivation domain (TAD) located from amino acids 486–691 are required for the transcription of viral genes and viral reactivation [146]. The transactivation domain deficient Rta mutant (Rta∆STAD) including amino acids 1–530 was shown to be unable to activate the transcription of the Mta promoter alone, and upon co-transfection with WT Rta inhibited activation by WT Rta in a dose-specific manner. Furthermore, Rta∆STAD was unable to induce reactivation as measured by the expression of markers K8.1 and ORF59 in KSHV-infected B cells. In addition, in B cells transfected with Rta∆STAD and treated with the viral inducer TPA, a noticeable decrease in the expression of K8.1 and ORF59 was observed, indicating that not only is Rta∆STAD reactivation deficient, but also forms inactive multimers, thus regulating Rta transactivation in a dominant-negative fashion. Co-immunoprecipitations performed both in vivo and in vitro confirm this homodimerization of Rta∆STAD and WT Rta, as WT Rta was detected in samples immunoprecipitated using antibodies specific for Rta∆STAD [129,151]. Further co-immunoprecipitations were performed using differentially tagged WT Rta, confirming that full length Rta forms homomultimers. Gel filtration chromatography proved that these complexes include Rta tetramers, and Rta mutants that exclusively form tetramers retain WT transactivation and reactivation functions [151].

Because the deletion of the leucine rich region of Rta prevented Rta∆STAD from competing with full length Rta for transactivation, this region was implicated in participating in Rta:Rta interactions. To further confirm this finding and identify the domains required for homomultimerization, truncated Rta mutants ranging from amino acids 1–238, 1–272, and 1–414 were generated, and co-immunoprecipitations performed for each with WT Rta [151]. Although the mutant containing amino acids 1–272 contained the leucine rich region, only truncated Rta containing amino acids 1–414 was precipitated by WT Rta, indicating that the leucine rich region alone was not sufficient for Rta:Rta interactions. Furthermore, this data shows that amino acids 238–414 are necessary for Rta to bind to itself.

In addition to binding itself, Rta also binds the cellular Notch signaling pathway protein, RBP-Jk, an interaction that is also necessary for the transactivation of viral genes and reactivation [147,152]. In the canonical model for Notch, RBP-Jk functions as a transcriptional repressor in the absence of Notch activation [153]. However, activated Notch binds to RBP-Jk to specify genes for RBP-Jk-dependent transactivation. Therefore, the direct interaction between Rta and RBP-Jk is functionally similar to that of Notch intracellular domain; i.e., activated Notch (NICD) and RBP-Jk. However, unlike NICD’s stable interaction with a single domain of RBP-Jk, two independent domains of RBP-Jk, amino acids 1–180 and 179–500 of RBP-Jk, were each necessary and sufficient to bind to Rta. These data suggest that Rta tetramers might contact multiple domains of RBP-Jk simultaneously, one potential explanation for the functional differences observed between Rta and NICD. Moreover, unlike the canonical model for Notch (see Section 3.1, below), there is no formal evidence that Rta displaces transcriptional co-repressors from DNA bound RBP-Jk. Instead, current evidence supports the conclusion that RBP-Jk specifies only some of Rta’s transcriptional targets; indeed, RBP-Jk is not poised uniformly as a transcriptional repressor on the KSHV genome, but rather Rta stimulates RBP-Jk binding to a subset of viral promoters [13,154].

To determine the region of Rta necessary for binding RBP-Jk, various Rta truncations were made and GST pull-downs performed to determine the regions necessary for Rta:RBP-Jk complex formation. Amino acids 170–400 of Rta were found to be necessary for Rta:RBP-Jk complex formation. Furthermore, this region contains the leucine rich repeat region, which, when deleted, severely compromises Rta:RBP-Jk complex formation, indicating that the entirety of this region is necessary for Rta:RBP-Jk interactions.

In addition to domains required for Rta’s transactivation function, portions of Rta play a role in Rta’s ability to function as a ubiquitin E3 ligase and to contribute to replication of the viral genome. Mutations made within the 118–207 amino acid region of Rta rendered Rta unable to degrade both nuclear and cytoplasmic IRF7, indicating that this region is essential for this function [155], while amino acids 401–500 contribute to the interaction with ORF59 and viral replication [134]. IRF7 is a transcription factor that mediates type I IFN induction [156]. As such, the regulation of IRF7 degradation allows Rta to also control IFN expression.

**Figure 5:**
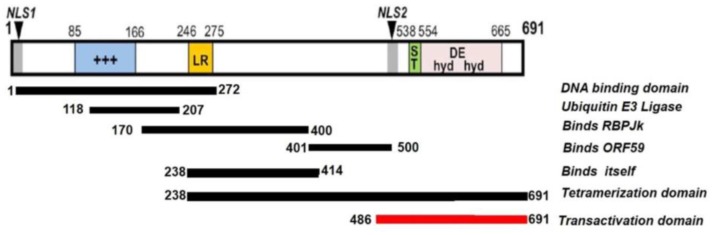
Protein structure of replication and transcriptional activator (Rta). The Rta protein is 691 amino acids and includes two nuclear localization signals (black arrows/gray boxes), a serine/threonine rich region (green rectangle), a basic amino acid rich region (blue), a leucine heptapeptide repeat domain (yellow), and a region of hydrophobic and acid amino acid repeats (pink). Figure and legend modified with permission from [157].

A summary of these domains and their functions is indicated in (Figure 5).

### 2.2. Functions

The structure of Rta provides insight into its function, with most of Rta’s domains playing a role in its transactivation and reactivation abilities. However, Rta’s structure also indicates that Rta is directly involved in vDNA replication and protein degradation.

#### 2.2.1. Viral DNA Replication

Eight viral proteins, ORF59, ORF6, ORF40/41, ORF44, ORF56, ORF59, K-bZIP, and Rta are required for the replication of the KSHV genome at the origin of replication [158]. Rta, specifically, contributes to viral replication through its interaction with the processivity factor ORF59. ORF59 is brought to the nucleus by ORF6 [159], where it is phosphorylated by ORF36 [160]. This phosphorylation event is required for the interaction of ORF59 and Rta [160]. Consequently, interaction with Rta is required for ORF59 binding to the origin of replication as well as the initiation of replication [134]. As such, Rta plays an essential role in directly regulating the replication of the viral genome.

#### 2.2.2. Transactivation of Viral Promoters

Rta’s most well-known and direct contribution to viral reactivation is through the transactivation of viral genes [129]. Rta has been shown to transactivate 34 viral promoters in uninfected cells [143], including: vIL6, nut-1/PAN, ORF57/Mta, ORF59, K-bZIP, vIRF1, ORFK1, ORF65, ORF56, SOX (ORF37), vOX, and ORF52 [149]. In addition to these promoters, Rta has been shown to regulate its own expression through the transactivation of the Rta promoter [149].

To determine the mechanism by which Rta activates these promoters, thus contributing to viral reactivation, many studies were performed to identify the components of the Rta transactivator complex. Screens for potential Rta-interacting proteins were performed using a yeast two-hybrid approach, which identified the cellular protein, RBP-Jk. Co-immunoprecipitations confirmed the RBP-Jk–Rta interaction [152]. To determine whether Rta required RBP-Jk to transactivate viral genes, viral promoters were transfected into RBP-Jk null cells and transactivation by Rta alone or Rta and RBP-Jk analyzed. Rta alone was unable to activate the transcription of these viral genes, but was able to do so when RBP-Jk was added, indicating that RBP-Jk is necessary for Rta transactivation of some viral genes [152,161]. Furthermore, transactivation deficient Rta (Rta∆STAD) is sufficient to rescue transactivation by RBP-Jk fused to a VP16 activation domain, and stimulates RBP-Jk DNA binding [13]. The requirement of RBP-Jk for mature virion production was confirmed by inducing the lytic cycle in RBP-Jk null cells [162]. Rta alone was insufficient to induce the expression of K8.1, K-bZIP, and ORF59, but was able to induce the expression of these markers in RBP-Jk positive cells [147]. Electromobility shift assays identified that Rta:RBP-Jk complexes recognize RBP-Jk sites on some viral promoters through binding to CANT consensus sites located near RBP-Jk binding sites [13,154]. CANT sites all contain the core tetranucleotide sequence CAnT and conform to the consensus sequence 5′-ANTGTAACANT(A/T)(A/T)T-3′ in the KSHV genome.

**Figure 6:**
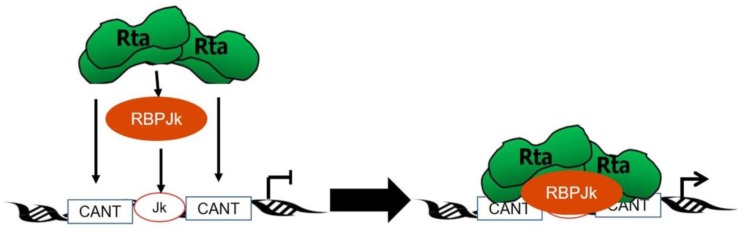
Model of viral transcription and reactivation by Rta. In the current model of viral reactivation, Rta (green) forms a tetramer and then complexes with cellular RBP-Jk (orange oval). This complex then recognizes RBP-Jk binding sites (orange outlined boxes) located within viral promoters through binding to CANT DNA repeats (blue outlined boxes). Once bound, the Rta:RBP-Jk complex activates the transcription of viral genes, thus leading to viral reactivation. Adapted with permission from [154].

It was further discovered that Rta and RBP-Jk do not form a complex in a 1:1 ratio, but rather that Rta forms a tetramer. Rta mutated to ensure tetramer formation was able to activate the transcription of the K-bZIP viral promoter at levels similar to that of wild-type Rta [151]. This information, taken together with the knowledge that RBP-Jk is required for viral reactivation, provides the current model of viral transcription and reactivation mediated by Rta (Figure 6).

While RBP-Jk is necessary for the Rta transactivation of some viral genes, Rta transactivates other viral promoters in an RBP-Jk-independent fashion [152,163]. Moreover, many studies suggest that additional molecular interactions are essential for the formation of a transcriptionally competent Rta-RBP-Jk complex [13,154,164,165,166]. These interactions include the direct binding of Rta to DNA (Figure 6) and to the cellular protein Octamer-1 [164]. Indeed, a DNA binding mutant of Rta has been described that retains its ability to bind RBP-Jk but is reactivation deficient [166]. Data from that publication also demonstrated that Rta can sequester RBP-Jk to disrupt normal Notch signaling.

#### 2.2.3. Degradation of Cellular Proteins

Rta has been shown to have ubiquitin E3 ligase activity, which targets IRF7 for degradation via the ubiquitin proteasome pathway. Increasing amounts of Rta decrease the amounts of IRF7 present in a dose-dependent fashion. Upon treatment with a proteasome inhibitor, this decrease was no longer observed. In addition, when cells were co-transfected with DNA for IRF7 and ubiquitin, ubiquitinated IRF7 was detected in samples lacking Rta. However, upon the addition of Rta, both ubiquitinated IRF7 and unmodified IRF7 levels were reduced, indicating that Rta functions as a ubiquitin E3 ligase capable of degrading IRF7. Furthermore, Rta mutants were unable to inhibit the accumulation of IRF7 in 293 cells [155]. It was also shown that Rta was capable of degrading itself via this pathway [155]. Not surprisingly, further studies have shown that Rta can degrade a number of different proteins via this pathway, including the transcription factor and known Rta repressor, NF-κB [167]. However, further analysis indicates that this downregulation of NF-κB is not direct, but rather regulated through the targeting of vFLIP by Rta for degradation [168]. Further cellular proteins regulated via Rta-mediated degradation include the downstream target of the Notch signaling pathway, Hey 1 [169]. Degradation of Hey 1 by Rta allows Rta to regulate its own expression, as Hey 1 can function as an Rta repressor [169]. Rta also upregulates Hey 1 expression [170], further supporting the hypothesis that Rta tightly regulates KSHV reactivation.

Rta’s ability to manipulate Hey 1 expression to act as a feedback loop, coupled with the requirement of RBP-Jk for the transactivation of viral genes, indicates a complex relationship between Rta and the Notch signaling pathway.

## 3. Notch

The Notch signaling pathway is a highly conserved mechanism used to regulate gene transcription in response to extracellular ligands. The effects of Notch pathway dysregulation were first observed in female *Drosophila* containing a mutant Notch allele [171]. Female mutants with only one wild-type copy of this gene were found to exhibit serrations or “notches” in their wing tips. Furthermore, this gene was shown to be X-linked in *Drosophila*, as upon cross-breeding with wild-type males, none of the surviving male progeny exhibited this phenotype.

Since 1917, when the first observation about Notch was made, more insights into the components of the Notch signaling pathway, Notch’s role in cell signaling, and its importance to cell regulation have been made. In fact, Notch dysregulation has been implicated in developmental syndromes [8,9] and adult onset diseases (reviewed in [8]), and Notch can function as on oncogene in numerous cancers such as T cell acute lympohoblastic leukemia/lymphoma [172], melanoma [173], breast cancer [174], and ovarian cancer [175].

### 3.1. Function

In its ground state, the Notch protein component of the Notch signaling pathway functions as a transmembrane receptor. As such, the epidermal growth factor (EGF) domain repeats 11 and 12 of the Notch extracellular domain recognize Delta (DLL)- and Jagged (JAG)-like ligands [176] located on the surface of neighboring cells. These ligands contain Delta, Serrate, and Lag2 (DSL) and EGF domains, which are required for interacting with Notch [177]. This interaction leads to a series of conformational changes allowing the Notch heterodimerization domain (HD) to be cleaved by A Disintegrin and Metalloprotease (ADAM) twelve amino acids before the Notch transmembrane domain. This cleavage event releases the Notch extracellular domain and creates a membrane-tethered intermediate. The Notch intermediate is then cleaved by γ-secretase within the transmembrane domain (reviewed in [178,179]), thus releasing the Notch intracellular domain (NICD). Because of its role in the final activation of the Notch receptor, gamma secretases are popular targets of Notch inhibitors [12,172].

**Figure 7:**
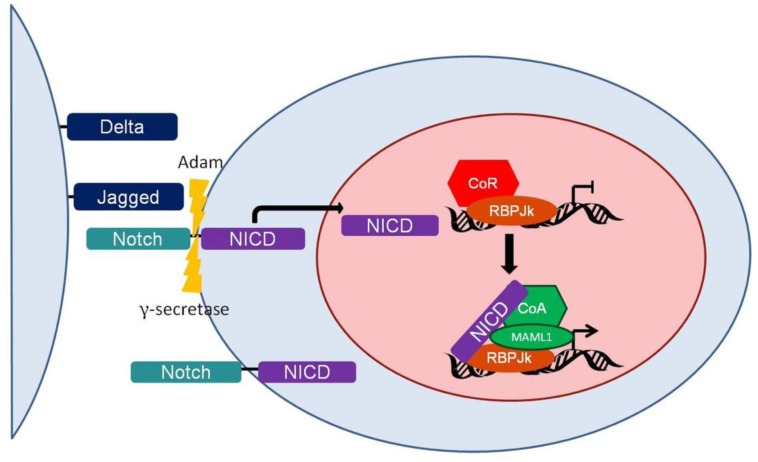
The canonical Notch signaling pathway. Jagged- and Delta-like ligands (dark blue) expressed on the surface of neighboring cells induce the proteolytic cleavage of the Notch extracellular domain (teal) from the intracellular domain (purple) by A Disintegrin and Metalloprotease (ADAM) and γ-secretase (lightning bolts). Following cleavage, the intracellular domain translocates to the nucleus where it associates with RBP-Jk (orange circle) to release the transcriptional co-repressors (red hexagon) and recruit co-activators (green hexagon and circle). These co-activators change the epigenetic landscape, allowing transcription to occur. NICD, Notch intracellular domain.

Once released from the membrane, NICD translocates to the nucleus where it interacts with RBP-Jk bound to DNA. In the absence of Notch, RBP-Jk is bound by transcriptional co-repressor complexes containing the proteins SHARP/MINT, Drosophila Hairless, KDM5A histone demethylase, or KyoT2 [153,180]. These proteins recruit histone deacetylases to prevent transcription. When NICD associates with RBP-Jk, the co-repressors are released, and transcriptional co-activators are recruited. These co-activators include mastermind/mindbomb (MAML) and histone acetyltransferases (HAT). MAML stabilizes the RBP-Jk:NICD:DNA complex to recruit the histone acetyltransferases and change the epigenetic landscape to activate transcription [181]. Notch pathway induction activates the expression of many cellular genes, including: the transcription factors Hes1, Hes5, Hey1, cMYC, and GATA3; cyclin D1, a regulator of cell cycle progression; and Notch isoforms 1 and 3 (reviewed in [153]), indicating the importance and complexity of the Notch signaling pathway. Furthermore, the amount and types of genes transactivated by Notch are both cell-type and Notch isoform-dependent [182]. The Notch signaling pathway is summarized in Figure 7.

### 3.2. Structure

To fully understand the role of Notch in cell signaling, it is first important to understand its structure. The 2543 amino acid mammalian Notch protein consists of two main domains: the extracellular and intracellular domains. The extracellular domain contains regions required for ligand recognition, while the intracellular domain contains regions required for transcriptional activation and self-regulation.

The Notch extracellular domain is composed of the conserved epidermal growth factor (EGF) domain and the negative regulatory region (NRR), consisting of the Lin-12-Notch repeats (LNR) and heterodimerization domain (HD). The EGF domain consists of 36 EGF repeats [183]. Of these 36 repeats, only repeats 11 and 12 are required for interaction with Jagged and Delta-like ligands [176], and repeats 24–29 may be involved in Notch inhibition by ligands expressed in the same cell, such as calcium ions [176,184]. While the EGF domain is involved in ligand interaction, the negative regulatory region ensures that the activation of Notch does not occur without this interaction. The LNR of the negative regulatory region wraps around the heterodimerization domain, protecting it from cleavage events that would activate the protein [185]. Activating mutations within this domain were discovered in T-ALL, resulting in constitutively active Notch [186].

The crystal structure of the Notch-Jag1 and Notch-DLL4 complexes reveal that Notch EGF repeats 8–12 are required for Notch-Jag1 interactions, while EGF repeats 11–13 are required for interaction with DLL4 [187]. Furthermore, it was shown that DLL4 has a higher affinity for Notch binding than Jag1. However, extended binding between Notch and the ligand is required to ensure conformational changes remain in place long enough to allow the cleavage of the extracellular domain from the intracellular domain. Jag1 has been shown to undergo hinge-like movements that extend the bond time, allowing for downstream cleavage events.

The Notch intracellular domain (NICD) consists of the transmembrane, RBP-Jk association module (RAM), proline/glutamic acid/serine/threonine-rich (PEST) domains, and ankyryn-like repeats. The transmembrane domain spans the cellular membrane, connecting the domains required for ligand recognition and those involved in transactivation. This domain is largely alpha helical in structure [188]. The RAM and ankyryn-like repeat domains are required for interaction with the cellular repressor, recombination signal binding protein Jk (RBP-Jk)/CBF-1, Su(H), Lag-1 (CSL) [189]. More specifically, the RAM domain directly interacts with the β trefoil domain (BTD) of RBP-Jk, while the ankyryn domain acts as a site for the binding of the RBP-Jk C terminal domain and N terminal domain of Mastermind/Mindbomb (MAML), a protein that forms part of the activation complex [181].

The crystal structure for the Notch:RBP-Jk:Mastermind complex reveals an N terminal cap structure directly preceding the ankyryn repeat region that likely stabilizes the interaction between Notch and RBP-Jk [181]. Furthermore, the beta hairpin loop within the ankyryn domain of Notch has been shown to interact with the BTD of RBP-Jk. Previous studies of the crystal structure of RBP-Jk noted that the BTD domain was lacking a β hairpin motif typically found in a β trefoil fold [190]. However, binding of the RAM domain of Notch to this region mimics the missing β hairpin motif. It is also of note that the Notch RAM domain contains the ΦPΦW motif, which is conserved in the EBV transactivator EBNA2, but not in Rta [191]. This motif is the most specific determinant of RBP-Jk binding.

The transactivation domain harbors the PEST domain, which is responsible for the stability of the Notch intracellular domain. Numerous studies have discovered mutations within this domain in cancer derived cells, all of which have been found to increase the stability of Notch [186,192]. Current studies indicate that phosphorylation of the PEST domain recruits the E3 ubiquitin ligase complex for NICD degradation [193]. The four Notch isoforms 1–4 all contain conserved domains within the extracellular and intracellular domains apart from the transactivation domain. The transactivation domains of Notch 3 and 4 contain the PEST domain, but lack the phosphorylation sites that allow for the modulation of Notch activity by other cellular signaling pathways [194,195].

**Figure 8:**
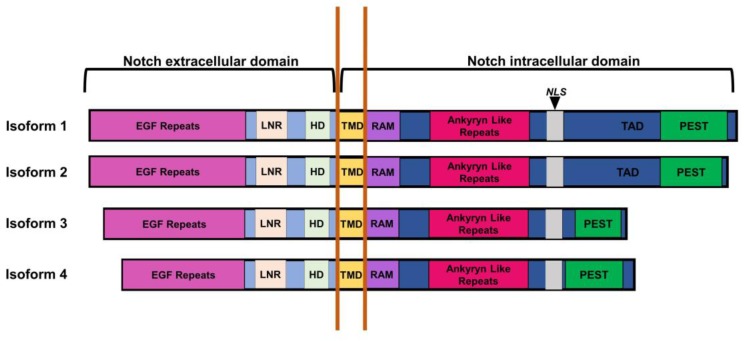
Summary of Notch’s secondary structure. The four Notch isoforms each consist of an extracellular and an intracellular domain (indicated by black bars). The extracellular domain comprises epidermal growth factor (EGF)-like repeats (pink box), lin-12 Notch repeats (light pink square), and the heterodimerization domain (light green square). While these domains are highly conserved, the four Notch isoforms differ in the number of EGF repeats (29–36). The components of the extracellular domain all play a role in ligand recognition and Notch activation. The intracellular domain of Notch consists of the transmembrane domain (yellow box) which spans the cellular membrane (indicated by orange lines), RBP-Jk association module (RAM) (purple box), ankyryn (magenta box), and proline/glutamic acid/serine/threonine-rich (PEST) (green box) domains. The RAM and ankyryn domains play a key role in interacting with the transcriptional repressor, RBP-Jk, and recruiting other coactivators to the protein complex, while the PEST domain regulates Notch stability. The NICD also contains a nuclear localization signal (gray box), which directs NICD for transport into the nucleus. NICD 1 and 2 both contain a transactivation domain (TAD) that may allow other pathways to regulate Notch activity.

Notch’s secondary structure and domain composition are summarized in Figure 8.

### 3.3. Dysregulation in KSHV Infection

As discussed, the KSHV protein Rta transactivates viral genes to induce viral reactivation in a mechanism that requires the Notch signaling component RBP-Jk [147,152]. The dysregulation of the Notch signaling pathway by Rta coupled with Notch dysregulation in cancer suggested that other components of the Notch signaling pathway, such as Notch itself, might be dysregulated in KSHV as well.

#### 3.3.1. Notch in KSHV Disease Progression and Oncogenesis

The initial studies in KSHV-infected cells noted elevated Notch expression. Immunostaining of KS tumor cells showed increased cytoplasmic staining for Notch isoforms 1 and 4. Perinuclear Notch staining and increased expression of the Notch target, Hes-1, indicated that Notch isoforms 1 and 4 were active [6]. No increase in cytoplasmic Notch isoform 2 was observed in KS tumor cells, but an increase in nuclear and perinuclear Notch 2 was observed. Further studies performed in KSHV-infected lymphatic endothelial cells detected an increase in the expression of the Notch ligands DLL4, JAG1, and DLL3, the Notch targets, Hes-1 and Hey-1, and Notch receptor 4 via mRNA levels [10]. These results indicate that Notch is in fact overexpressed and active in KSHV-infected cells, implicating Notch signaling in KS progression and tumor development.

Multiple KSHV proteins contribute to Notch receptor overexpression and constitutive activation. In lymphomas from Eμ-v-cyclin mice, wherein KSHV v-cyclin initiates T cell lymphoma, increased expression of Notch3 was observed [196]. In uninfected 293 cells, the ectopic overexpression of the viral lytic protein vGPCR and vIL6 induces the expression of Notch2 and 3, and Jagged 1, and the overexpression of vIL6 induces the expression of Notch4, DLL1, and DLL4, respectively [197]. Furthermore, the latent protein vFLIP induced increases in Notch 1 and 4 expression as well as the DLL1 and 4 ligands, while LANA increased DLL4 expression. In a similar model, LANA increased Notch activation both by inducing DLL4 expression and stabilizing activated Notch. In the absence of LANA, Sel10 directly binds to NICD and targets it for degradation [193]. LANA increases NICD stability by binding the WD40 domain of Sel10, thus directly competing with NICD for Sel10 binding [198]. In addition to increasing NICD stability, LANA also prevents the degradation of the NICD target, Hey1 [199]. LANA-dependent Hey1 accumulation was shown to be necessary for angiogenesis.

To determine the pathologic consequences of increased Notch receptor expression and activation on KSHV infection and disease progression, labs have evaluated the effects of treating cells with gamma secretase inhibitors (GSI) such as *N*-[(3,5-Difluorophenyl)acetyl]-l-alanyl-2-phenyl]glycine-1,1-dimethylethyl ester (DAPT) [6,12]. The inhibition of Notch by gamma secretase inhibitors in infected SLK cells not only reduced the activation of Notch isoforms 1, 2, and 4 and the downstream targets Hes1, Hey 1, and Hey 2, but also induced apoptosis [6]. The same effect of GSIs was observed in primary KS tumor cells [6] and infected PEL cells [11]. In lymphomas induced in nonobese diabetic/severe combined immunodeficiency (NOD/SCID) mice inoculated with KSHV infected PEL cells, treatment with GSI resulted in the necrosis of the lymphomas [12], thus supporting the hypothesis that Notch signaling was essential for disease progression in both cultured cells and animal models.

Because of Notch’s overexpression in KSHV-infected cells and tumors, coupled with the apoptosis and necrosis observed in GSI treated samples, many labs have attempted to determine the mechanism by which Notch might contribute to KSHV progression. Multiple studies have shown a link between Notch signaling and the endothelial to mesenchymal transition (EMT), a step considered to be a hallmark of KSHV infection [7,197,200].

Lymphatic endothelial cells (LEC) infected with KSHV express endothelial precursor markers such as CD133 and late mural cell markers [197]. In these cells, both latent and lytic viral proteins, including Rta, increased the expression of Notch ligands and receptors. The inhibition of Notch using GSI and soluble DLL4 decreased the expression of the endothelial precursor marker, CD133. This was the first evidence that Notch plays a role in EMT in KSHV-infected cells.

Additional studies showed that DAPT treatment of KSHV-infected LECs decreased sprouting, a key EMT process [7]. Moreover, Notch inhibition also downregulated the mesenchymal markers PDGFRB, TAGLN, VIM, and CDH2. Inhibiting the viral proteins vGPCR and vFLIP also decreased sprouting, suggesting that these proteins regulate Notch in KSHV-infected cells. In this cellular pathogenesis model, the viral proteins vGPCR and vFLIP influence Notch expression, leading to the activation of Membrane-Type-1 Matrix Metalloproteinase (MT1-MMP) expression, an enzyme involved in cancer cell invasion, to affect EMT.

Building upon these studies, Notch was also shown to stimulate the EMT in KSHV-infected dermal microvascular endothelial cells (DMVEC) [200]. Again, treatment with DAPT resulted in a decrease in the expression of the mesenchymal markers and transcription factors involved in EMT, called Slug, ZEB1, and ZEB2. These proteins were thus all shown to be overexpressed in a KSHV- and Notch-dependent manner.

These three studies conclusively show that KSHV stimulates the EMT in a Notch-associated manner. Each of these studies implicated different mechanisms by which Notch regulated this transition, highlighting a primary role for Notch in disease progression in KSHV-infected cells [7,197,200].

Other studies suggest that Notch isoform 1 drives oncogenesis in KSHV infected cells, as its inhibition leads to necrosis [11]. KSHV-infected B cells with high Notch expression levels exhibited greater proliferation than B cells with low Notch levels. The inhibition of Notch resulted in cell cycle arrest at G1 and a decreased expression of cyclin D1, which promotes progression through the G1/S phase. The cyclin D1 promoter was activated by NICD 1, indicating that Notch also contributes to disease progression by driving cell proliferation and thus oncogenesis by activating cyclin D1 transcription.

#### 3.3.2. Notch in KSHV Reactivation

Based on the knowledge that there are approximately 81 consensus and additional non-consensus RBP-Jk binding sites within the KSHV genome [32] (Lukac Lab, Unpublished observations), and that the majority of cells in a KS tumor are latently infected, it is surprising that constitutively active Notch would be detected in KSHV-infected cells and be so vital to infected cell survival. One would reasonably expect constitutively active Notch to initiate viral gene transcription and reactivation and subsequent cell death. However, the effect of activated Notch on the virus is not straightforward.

One of the earliest studies to address the effect of Notch on KSHV showed that Notch can activate viral gene expression. The Jung lab expressed doxycycline-inducible NICD 1 in infected PEL cells, and identified a subset of viral genes from all points during the lytic and latent cycles of KSHV that were activated [201,202]. However, NICD 1 was not able to induce the expression of all viral genes. It is of note that one of the delayed early genes induced by NICD 1 overexpression, ORF57, was shown by our lab and others to enhance reactivation by Rta [135,136]. Further studies from our lab show that NICD 1 cannot directly transactivate the Mta promoter in uninfected cells unless Rta stimulates RBP-Jk binding to the promoter [13]. Taken together, these data suggest that Notch may regulate gene-specific KSHV expression in a manner that differs in the presence or absence of Rta.

As the literature had already voluminously demonstrated that Notch also activates the expression of repressed EBV viral genes in an RBP-Jk-dependent fashion [203,204,205], we and others asked if Notch was capable of productively reactivating KSHV from latency. Using luciferase assays, it was shown that the intracellular domain of Notch 1 in complex with RBP-Jk is capable of activating the Rta promoter [15], providing further evidence that Notch isoform 1 might play a critical role in viral reactivation. Using immunofluorescence of both Rta and another viral protein LANA, coupled with fluorescence-activated cell sorting (FACS) analysis and extracellular viral DNA amplification via PCR, it was shown that expression of ectopic NICD 1 is sufficient to induce viral reactivation in infected B cells [15]. However, our lab reported that NICD 1 was unable to reactivate KSHV in PEL cells as measured by immunofluorescence of the true late gene, K8.1, viral extracellular DNA amplification using real time PCR, or Southern blotting [13], nor through the induction of the infectious reporter virus from Vero cells [14]. Ectopic NICD2, 3, and 4 are similarly unable to induce KSHV reactivation [14].

Further complicating matters, a recent study in infected SLK cells provides evidence suggesting that Notch does not positively contribute to viral reactivation, but instead inhibits reactivation [16]. Using infected SLK cells treated with a known viral inducer and the Notch inhibitors, DAPT and Notch1 specific siRNAs, this study shows an increase in the mRNA expression of viral lytic genes when Notch activity or expression is reduced [16]. The proposed mechanism for this action is through the direct inhibition of the Rta promoter by the Notch downstream effector, Hes1.

However, we could not corroborate those data in a new latency/reactivation system that measures the production of infectious virus as detected by a trans-complemented reporter. These infected Vero cells permit the efficient transfection of plasmid DNA and siRNA, and yield robust and reproducible reactivation. In that work, we inhibited Notch through the use of DAPT, siRNAs, and the ectopic expression of a dominant negative mastermind mutant [206], all of which decreased the amount of infectious virus produced in response to HDACi or ectopic Rta (Lukac Lab, Unpublished).

To confirm these observations in human cells, we treated PEL cells with the viral inducer, valproic acid (VPA), and Notch inhibitor, DAPT, and scored reactivation based on the expression of the late gene K8.1. Using immunofluorescence, we observed a decrease in K8.1 expression upon treatment with DAPT (Lukac Lab, Unpublished), but no decrease in Rta expression (Lukac Lab, Unpublished).

All of these provocative, yet contradictory results hint at the complexity of interactions of Notch with KSHV and demand further studies to fully understand the effects of Notch on viral infection.

## 4. Conclusions

Many viruses manipulate host signaling pathways to regulate viral gene expression. KSHV employs this biologic strategy by manipulating the Notch pathway. Due to the presence of RBP-Jk sites within lytic gene promoters [32] and Notch’s functional homology to the lytic switch protein, Rta, it is unsurprising that a number of researchers have proposed that Notch may have a role in regulating KSHV reactivation [13,15,202]. Indeed, engaging Notch activity downstream of the viral switch protein Rta facilitates lytic gene expression (Lukac Lab, Unpublished). This observation implies a scenario in which Notch could activate viral productive cycle genes without the need for continued Rta antigen expression. In turn, this scenario could be consistent with KS progression.

Studies reporting the overexpression of activated Notch in KS lesional tissue and infected cells [6] corroborates well with the data from other cancers, including T-ALL, that indicate a primary role for constitutive Notch activity in tumor growth and malignancy [186]. Furthermore, studies show that the inhibition of Notch induces apoptosis in KSHV-infected cells [6] and contributes to a decrease in tumor size in infected mice [12] as is observed in other non-viral cancers [207].

The contribution of the Notch signaling pathway in KSHV disease progression remains a vital topic in virology. A consensus on whether Notch is sufficient to “flip the switch” and reactivate KSHV from latency has not yet been reached. Conflicting answers to that question support potential cell-specific effects for Notch activity in reactivation. Because the literature also reports promoter-specific effects of activated Notch for the virus and host cell, the means of quantitating reactivation could also influence the conclusions of different studies. Superficially, Rta appears to mimic activated Notch, but detailed studies highlight significant mechanistic differences between the proteins. Continued investigation of the mechanism by which Notch and Rta function in B cells during KSHV reactivation is required to grasp the complexity and pathologic consequences of KSHV–Notch interactions and regulation.
